# *In vitro* analysis of the effects on wound healing of high- and low-molecular weight chains of hyaluronan and their hybrid H-HA/L-HA complexes

**DOI:** 10.1186/s12860-015-0064-6

**Published:** 2015-07-11

**Authors:** Antonella D’Agostino, Antonietta Stellavato, Teresa Busico, Agata Papa, Virginia Tirino, Gianpaolo Papaccio, Annalisa La Gatta, Mario De Rosa, Chiara Schiraldi

**Affiliations:** Department of Experimental Medicine, Section of Biotechnology, Medical Histology and Molecular Biology “A. Cascino”, Second University of Naples, Via De Crecchio 7, 80138 Naples, Italy; Novartis Vaccines and Diagnostics Srl, Via Fiorentina 1, 53100 Siena, Italy; ALFATESTLAB s.r.l, Via Pelizza da Volpedo,, 59 20092 Cinisello Balsamo, MI Italy

**Keywords:** Wound healing, Hyaluronan, MMPs, Hybrid complexes

## Abstract

**Background:**

Recent studies have reported the roles of Hyaluronic acid (HA) chains of diverse length in wound repair, especially considering the simultaneous occurrence *in vivo* of both high- (H-HA) and low-molecular weight (L-HA) hyaluronan at an injury site. It has been shown that HA fragments (5 ≤ MW ≤ 20 kDa) usually trigger an inflammatory response that, on one hand, is the first signal in the activation of a repair mechanism but on the other, when it’s overexpressed, it may promote unwanted side effects. The present experimental research has aimed to investigate H-HA, L-HA and of a newly developed complex of the two (H-HA/L-HA) for stability (*e.g.* hyaluronidases digestion), for their ability to promote wound healing of human keratinocytes *in vitro* and for their effect on cellular biomarker expression trends.

**Results:**

Time-lapse video microscopy studies proved that the diverse HA was capable of restoring the monolayer integrity of HaCat. The H-HA/L-HA complex (0.1 and 1%w/v) proved faster in regeneration also in co-culture scratch test where wound closure was achieved in half the time of H-HA stimulated cells and 2.5-fold faster than the control. Gene expression was evaluated for transformation growth factor beta 1 (TGF-β1) proving that L-HA alone increased its expression at 4 h followed by restoration of similar trends for all the stimuli. Depending on the diverse stimulation (H-HA, L-HA or the complex), metalloproteinases (MMP-2, -9, -13) were also modulated differently. Furthermore, type I collagen expression and production were evaluated. Compared to the others, persistence of a significant higher expression level at 24 h for the H-HA/L-HA complex was found.

**Conclusions:**

The outcomes of this research showed that, both at high and low concentrations, hybrid complexes proved to perform better than HA alone thus suggesting their potential as medical devices in aesthetic and regenerative medicine.

## Background

Frequently called hyaluronan (HA), Na-Hyaluronate, has been extensively used for biomedical applications, including osteoarthritis treatment, tissue augmentation and ocular surgery, and as scaffold for tissue engineering [1]. In particular, it has been well established that HA is associated with tissue repair [2–5]. Although this macromolecule participates in many different biological processes during regeneration, detailed action mechanisms have still not been thoroughly unravelled.

Being involved in cell proliferation and migration, HA also provides important functions in wound healing: its contribution in these processes is associated with the hydrophilic and highly osmotic features of the macromolecule, which allow for the control of associated inflammatory processes and skin hydration. In fact, HA creates an environment that loosens cell anchorage to the extracellular matrix (ECM), facilitating cell migration and division [6].

HA produces different effects on the basis of its molecular weight [7]. In the earliest phase of wound healing (WH) *in vivo*, there is a sharp increase in HA right after injury: in particular, high molecular weight HA (H-HA) accumulates and binds fibrinogen that is fundamental for clot formation. Furthermore, an initial large amount of H-HA opens up tissue spaces, facilitating polymorphonuclear leukocyte (PMN) access to the wound site for removal of dead tissue, debris, and bacteria. Thereafter, in the inflammatory stage, there is an accumulation of low molecular weight HA (L-HA), which is generated from degradation of H-HA, inducing cytokine response and stimulating angiogenesis [8]. Many reports have studied the effects of exogenous H-HA or L-HA to obtain information on the role of HA in wound healing and to design innovative pharmaceutical/cosmeceutical formulations based on HA size [9–14].

A potential functional significance in the turnover of HA fragments during wound repair has also been suggested *in vitro* [15–17]. For example, the importance of L-HA (35–280 kDa) in propagating an inflammatory response (through activation of macrophages and induction of chemokines) has been well described by McKee and co-authors [18]. Similarly, other studies have shown that low- and intermediate-molecular weight HA (20–450 kDa) stimulated gene expression in endothelial and epithelial cells, macrophages and eosinophils [19–21].

Cutaneous wound healing consists in re-epithelization, which involves migration and proliferation of keratinocytes to cover the dermal surface. For this reason, many studies have used systems that simplify the complex mechanism of repair, such as the scratch-wound *in vitro* assay [13, 22, 23]. In addition, extensive molecular studies have been conducted to assess gene expression and signal molecules related to the re-epithelization process.

It is known that the healing of human skin wounds requires a complex interplay between cells and ECM. A number of events converges on the process and are controlled by: growth factors including transforming growth factor β1 (TGF-β1) and metalloproteases (MMPs) [24]. TGF-β1 is one of the inflammatory process trigger markers and has also been recognized as a key regulator of type I collagen expression [25]. MMPs elicit a pro-inflammatory response for cellular recruitment and degradation of ECM components, promoting migration during wound closure and remodelling. Therefore, MMPs are up-regulated during wound healing in epidermal cells, dermal cells, fibroblasts and blood cells in mammals [26].

In this context, the aim of this study was to compare activity of high and low molecular weight HA and hybrid H-HA/L-HA complex in view of their potential application in medical devices for tissue regeneration.

## Experimental

### Materials

High- (H-HA; MW 1400 ± kDa, Mw/Mn = 1.5, Intrinsic viscosity =21.5 dl/g) and low-molecular weight hyaluronic acid (L-HA: Mw = 90 ± 5 kDa, Mw/Mn = 1.7 ± 0.07 Intrinsic viscosity =2.4 dl/g) were kindly provided by Altergon s.r.l., Italy. Altergon HA is a fermentative HA from *Streptococcus equi* ssp. *equi,* at pharmaceutical grade (*e.g.* purity >95 %, water content < 10 %, EU/mg < 0.05). This ensured that the products are controlled according to the pharmacopeia. Briefly in collaboration with Altergon, LHA 90 kDa was obtained through heterogeneous hydrolytic reaction in acidic conditions. The hydrodynamic characterization was performed using the SEC-TDA (Size Exclusion Chromatography-Triple Detector Array) equipment by Viscotek (Lab Service Analytica, Italy).

The H-HA/L-HA complex (1:1 weight ratio) was produced in our laboratory following the procedure described in patent application WO2011EP65633 [27], which was modified to obtain solutions (32 g/l) suitable for use in cell cultures (*i.e.*, use of a physiological buffer solution, PBS). For all samples, pH and osmolality were measured in order to perform experiments in physiological conditions (*i.e.*, pH 7.0–7.4; osmolality 300 mOsm). Endotoxin concentration (EU/mg) was evaluated with the Limulus test, and solutions were used only when the titer as <1 EU/mg. All solutions were sterilized in an autoclave at 1 bar for 12 min at 120 °C.

Bovine testicular hyaluronidase, BTH (EC 3.2.1.35), essentially salt free lyophilized powder with a specific activity of 1160U/mg was purchased from Sigma-Aldrich (Milan, Italy) (cat. N. H3884, lot. N. 057K7014).

HaCaT cells (Istituto Zooprofilattico, Brescia, Italy), a spontaneously transformed non-tumorigenic human keratinocyte cell line and human dermal fibroblast (HDF), a generous gift of Prof Caraglia, were cultured in Dulbecco’s Modified Eagle Medium (DMEM) supplemented with 10 % (v/v) heat inactivated fetal bovine serum (FBS), 100 U/mL penicillin and 100 μg/ml streptomycin. All materials were purchased from Flow Laboratories (Milan, Italy). The cells were grown on tissue culture plates (Corning Incorporated, New York, USA), using an incubator with a humidified atmosphere (95 % air/5%CO_2_ v/v) at 37 °C. Collagen was purchased from Sigma, Aldrich (Milan, Italy).

## Methods

### Dynamic viscosity measurements of HA gels

The H-HA and H-HA/L-HA (1:1 weight ratio) solutions were prepared to final concentrations of 0.1 and 1 % w/v in DMEM–1 % FBS in order to have the same conditions used for the time-lapse experiments described in the successive section. In particular, 0.05, 0.5, 0.1 and 1 % w/v H-HA were obtained from stock solutions of 0.1, 1, 0.2 and 2 % w/v in PBS buffer, successively sterilized in autoclave and, respectively diluted 1:1 with DMEM 2 % FBS. Analogously, 0.1 and 1 % w/v H-HA/L-HA complex gels were obtained from a 32 g/l stock solution sterile syringe (used as medical device) and opportunely diluted with the medium supplemented with final FBS 1%v/v. The dynamic viscosity (η_0_) of H-HA and H-HA/L-HA complex at the different concentrations was measured at 37 °C with a Paar–Physica oscillatory rheometer MCR 301 (Anton Paar, Germany) equipped with a coaxial cylinder cell. The η of each sample was measured in function of the shear rate (ranging from 0.001 to 300 s^−1^), the curves obtained evaluated and the value of dynamic viscosity (η_0_) chosen in the plateau of Newtonian behaviour.

### H-HA, L-HA and H-HA/L-HA degradation in the presence of BTH

The enzymatic degradation was carried as described by La Gatta et collaborators [28].

Briefly all the samples suspended at 0.4 % w/w in PBS were prepared and incubated in the presence of final concentration of BTH 0.5 U/ml at 37 °C in a thermomixer (Eppendorf, USA) under stirring (300 rpm). At increasing incubation times (1,2,4,6,8,24 h), samples were withdrawn from the thermomixer, boiled for 10 min to inactivate the enzyme and then stored at -20 °C until characterization. The chromatographic analyses of HA fragments produced during degradation were performed using the SEC-TDA equipment (on line laser light scattering, refractometry and viscosimetry) by Viscotek (Lab Service Analytica, Italy) [28]. Concerning our purposes, this analysis allowed us, to derive beside data such as Mw, Mn and polydispersity index, the fraction of samples with MW < 200 KDa, and the variation of this fraction during degradation was used as stability index respect to enzymatic hydrolysis.

### *In vitro* scratch-wound healing assay: cell culture and stimuli preparation

12-well tissue culture plates were collagen-coated with 100 μl of collagen type I (0.1 mg/ml in acetic acid) for 2 h before rinsing with complete medium. HaCat cells were seeded at a density of about 1.8 × 10^5^ cells/well (4 × 10^4^cells/cm^2^) in complete medium. Complete confluence was obtained after 2 days at 37 °C and 5 % CO_2_ (v/v). These standardized conditions were previously assessed on the basis of the growth curve for HaCaT cells (data not shown). Alternatively, in co-culture experiment, each 12-well was seeded with cells (HaCaT e HDF containing equal number of cells: 2.2 × 10^4^ cell/cm^2^). Scratch wounds were created mechanically with a sterile pipette tip (Ø = 0.1 mm). We were careful to produce uniformly sized wounds of approximately 0.5–0.9 mm in width. Detached cells and debris were washed away with PBS solution.

To test the effect of HA gels on the rate of wound closure, the scratched monolayers were incubated with 0.1 % w/v H-HA, L-HA and H-HA/L-HA in DMEM 1%FBS. Fresh serum-supplemented medium (1 % v/v FBS) was used as a control. The conditioned media were prepared as follows. Solutions of 1 % w/w H-HA/L-HA complex, H-HA or L-HA in PBS were prepared by diluting the stock solution (32 g/l in PBS) or directly dissolving the H-HA and L-HA powders. Solutions were then opportunely diluted in DMEM/FBS to have the correct final serum concentration established for the assay (1 % v/v). PBS buffer was used, instead of HA solution in PBS, for the control. In addition, the H-HA/L-HA complex was tested also at 1 % w/v by directly diluting the stock solution in the medium. Osmolality of all solutions was measured with an osmometer (Micro-osmometer automatic type 15Loser).

### Time-lapse video microscopy station: experimental set up

The ‘wound closure’ phenomenon was monitored for 72–96 h using a time-lapse video microscopy (TLVM), to observe and evaluate cell behaviour (adhesion, proliferation, differentiation and motility) (Okolab, Italy). The station is based on an inverted optical microscope (Zeiss Axiovert 200, Germany) equipped with a CCD–gray-scale camera (ORCA ER, Hamamatsu Photonics, Hamamatsu City, Japan), a microscope stage incubator (CO_2_, T and air control) that accommodates different wells, a thermostatic bath (LAUDA, Eco Line RE 204), and a remotely controlled motor that permits micrometric movements and the repositioning of the stage incubator along x, y and z cyclically over time. The CO_2_ microscope stage incubator maintains all the required environmental conditions for cell cultures (temperature and atmosphere were kept at 37 °C and 5 % CO_2_ in air, respectively). The instrument was controlled by the custom-tailored software OKO-Vision 4.3, composed of OKO-Vision Time Lapse and OKO-Vision Imaging. The former module is used to set the parameters of time-lapse experiments and to run the experiment by controlling the operations of the motorized stages; the latter is used to review the experiment by displaying the recorded images in different formats (panels and movies) and by quantitative analysis of wound healing. Measurements of wound closure was calculated as [(*Area t*_0_ − *Area t*)/*Area t*_0_] × 100 by the software. For images that were not clear the threshold was manually modified in order to better delineate the lesion area. Data generated by the software was exported in a common excel file and used to obtain the reduction in wound area (in squares micrometres) at each time-point. The reparation rate was then calculated as (*Areat*_1_ − *Area t*_2_/*t*_2_ − *t*_1_). Moreover, the fields of view selected and used to build up the overall averaged curves all had a similar scratch width, ranging from 0.7 to 0.9 mm, corresponding to a lesion area of 16–20 mm^2^.

The statistical significance of the experiment was ensured by the possibility to visualize contemporarily several fields of view (up to 24) of the same sample (depending on the delay time chosen by the operator) in the stage incubator. In our particular case, we collected phase-contrast images of wound margins every 30–60 min (*delay time*) throughout the experiment, until either healing closure was complete or no longer progressing. Furthermore, a single field of view (~10 × 10^5^ μm^2^) represented 5 % of the total scratch area (~20 × 10^6^ μm^2^) of each well. Because we captured at least five field-views in three repetitions per well, this ensured that we analysed 25–30 % of the scratch in each specific well. Triplicates were performed for each scratch assay.

### Real time PCR

To elucidate the mechanisms of wound closure promoted by HA, total RNA was extracted from control and stimulated HaCaT cells at different time-points. Specifically, after the insult (at least three scratches/well, corresponding to 30–40 % of the well area) and treatments, the cells were homogenized at different interval times (4, 10, 16 and 24 h) by TRIzol® (Invitrogen, Milan, Italy), and total RNA extracted according to the manufacturer’s instructions. The suspension, which was centrifuged and precipitated by the method mentioned above, was then re-suspended in nuclease-free water. The concentration of the extracted RNA was determined with a Nanodrop spectrophotometer (Celbio, Milan, Italy), and qualitative analysis of the RNA carried out through with 1 % agarose-gel (w/v) electrophoresis. For cDNA synthesis, performed with the Reverse Transcription System Kit (Promega, Milan, Italy), 1 μg of DNase-digested total RNA was used (DNA-free kit; Ambion-Applied Biosystems). Quantitative RT-PCR was obtained with the iQ™ SYBR® Green Supermix (Bio-Rad Laboratories s.r.l., Milan, Italy) to analyse the expression of TGF-β1, type I collagen, MMP-2, MMP-9 and MMP-13. BLAST query was used for the specificity analysis of each qRT-PCR primer pair and corresponding sequences designed by Beacon Designer™ software. The primer sequences and amplification protocol are given in Table 1. The final melting curve was performed from 55 to 95 °C. All reactions were carried out in triplicate, and the expression of specific mRNA relative to the control was determined after normalization with respect to the *HPRT* housekeeping gene, used as internal control [29]. The fold change in test mRNA expression was calculated by considering the efficiency of each primer pair (between 80 % and 110 %), and by using the comparative threshold method (2^∆∆Ct^) (∆∆Ct = difference of ∆Ct between treated cells and non-treated cells used as controls). The results were expressed as normalized fold expression, calculated by the ratio of crossing points of amplification curves of several genes and internal standard, using Bio-Rad iQ™5 software (Bio- Rad Laboratories s.r.l., Milan, Italy).

**Table 1:** Primers sequence used for qRT-PCR analysis

Gene	Forward primer	Reverse primer	Thermal Cycles
Transforming growth factor, beta 1 (TGFβ-1)	5′TgCggCAgCTgTACATTgA ′3	5′TggTTgTACAgggCCAggA′3	95 °C 10 s, 55 °C 30 s, 72 °C 3 min, 40 cycles
Collagen, type I, alpha 1 (COL1A1)	5′CAgCCgCTTCACCTACAg C′3	5′TTTTgTATTCAATCACTgTCTTgCC′3	94 °C 1 min, 56 °C 2 min, 72 °C 3 min, 40 cycles
Matrix metallopeptidase 2 (MMP-2)	5′gCCgCCTTTAACTggAgCAA′3	5′TTCCAggCATCTgCgATgAg′3	95 °C 10 s, 60 °C 30 s, 72 °C 3 min, 40 cycles
Matrix metallopeptidase 9 (MMP-9)	5′gCgCCACCACAgCCAACTATg′3	5′TggATGCCgTCTATgTCgTCTTTA′3	94 °C 1 min, 56 °C for 2 min, 72 °C 3 min, 40 cycles
Matrix metallopeptidase 13 (MMP-13)	5′TCCCAggAATTggTgATAAAgTAgA′3	5′CTggCATgACgCgAACAATA′3	95 °C 10 s, 55 °C 30 s, 72 °C 3 min, 40 cycles

### Gelatinase assay (MMPs -2 and -9)

In order to evaluate the presence of MMPs (-2 and -9) in the medium with and without the stimuli, an enzymatic assay was performed using gelatin as substrate.

A aqueous solution of commercial gelatin (Gelatin NITTA) 10 g/l was prepared; 100 μl of 24 h incubation cell medium deriving from a scratched monolayer, was added to 900 μl of gelatin substrate solution. The samples were untreated (CTR) and treated with H-HA 0.1 %, L-HA 0.1 % and H-HA/L-HA 0.1 % and 1 % respectively. Also a negative control consisting of the gelatin alone was incubated. The assay was performed at 37 °C under stirring; after 6 h the reaction was stopped by boiling the solution at 100 °C for 10′. The gelatin degradation was evaluated by HPLC-SEC analyses (UHPLC Dionex Ultimate 3000; Thermofisher), equipped with a UV-VIS detector (from 190 to 600 nm wavelength range). Runs were executed in isocratic mode on a Tosoh G2000WXL (7,8 × 300 mm, 5um) column and coupled to a precolumn Tosoh TSK Gel with an operating buffer containing NaCl 100 mM, filtered through a 0.22 μm membrane. Separations were performed at 20 °C, applying a flow of 0.5 ml/min for 40 min. The analysts were detected over the UV spectrum at 280 nm. Peak areas were evaluated through the Thermofisher Chromeleon Software. A gelatin standard curve (R^2^ = 0.9991) with five points (range 0.5–16 g/l) were used for quali-quantitative analyses.

### Gel zymography to evaluate activity of MMPs

Gelatin zymography was performed as follows: 10 % SDS-polyacrylamide separating gel with 0.2 mg/ml of gelatin (30 % bis-acrylamide, 1.5 M Tris-HCL pH 8.8, 10 % w/v ammonium persulfate, 0.04 % v/v TEMED, 10 % v/v SDS and distilled water to reach final volume) and 5 % SDS-polyacrylamide staking gel (30 % v/v bisacrylamide, 1 M tris-HCL pH 6.8, 10%w/v ammonium persulfate, 0.1 % v/v TEMED, 10 % v/v SDS and distilled water to reach final volume) was prepared. The gel was loaded with supernatants (20 μL) in non-reducing buffer (0.5 M Tris–HCl pH 6.8, 10 % v/v glycerol, 4 % v/v SDS, and 0.05 % w/v bromophenol blue) and the electrophoresis was carried at 4 °C at 90 V for approximately 2.5 h. Gels were washed 2 times in 2.5%v/v Triton-x 100 for 15 min, incubated on incubation buffer (Tris–HCl 50 mM, CaCl_2_ 10 mM, NaCl 50 mM, pH 7.6) for 16–18 h at 37 °C, stained by 1 % v/v Coomassie brilliant blue R-250 solution for 10 min under gentle shaking and then destained with 25 % v/v ethanol and 8 % v/v acetic acid solution. The gelatinase activity of each collagenase should correspond to a clear band against the blue background of stained gel.

### Protein extraction and western blot analysis

For protein extraction and Western blot analysis, HaCaT cells were lysed in a lysis buffer (Tris-HCl 10 mM, NaCl 150 mM, EDTA 2 mM, EGTA 2 mM, Triton X-100 4 %; Protease Inhibitor Cocktail (Sigma Aldrich, Milan, Italy)) 16 h after the scratch and treatments. Protein concentrations were determined with the Bradford method [30]. The samples were loaded, electrophorized on a 8 % SDS–polyacrylamide gel and electroblotted onto a nitrocellulose membrane. The membrane was blocked in 5 % milk, 1 % Tris-buffered saline and 0.05 % Tween-20. Primary antibody to detect MMP-13 (Santa Cruz Biotechnology Santa Cruz, CA, USA) was used according to the manufacturer’s instructions at a 1:100 dilution. Immunoreactive signals were detected with a horseradish peroxidase-conjugated secondary antibody (1:4000) and reacted with the ECL system (Chemicon-Millipore, Temecula, CA, USA). Protein levels were normalized with respect to the signal obtained with anti-α-tubulin monoclonal antibody (Santa Cruz Biotechnology, Santa Cruz, CA, USA; 1:1000). Semi-quantitative analysis of protein levels was obtained with the Gel Doc 2000 UV System and the Gel Doc EZ Imager, using quantity one software (Bio-Rad Laboratories s.r.l., Milan, Italy).

### Immunohistochemical analysis

Immunohistochemistry for type I collagen (Abcam, Cambridge, UK) was performed on scratched HaCaT cells in 12-well plates fixed with 4 % paraformaldehyde for 10 min at 4 °C and rinsed in PBS. For staining, the DAKO Cytomation En Vision + HRP kit AEC (DAKO, Milan, Italy) was used according to the manufacturer’s instructions. The type I collagen antibody was diluted 1:50 in PBS. Cells were observed using an inverted light microscope (Axovision zeiss 200). For Type I collagen quantification, TIFF images were analyzed with ImageJ software.

### Statistical analysis

The above described experiments were performed in duplicate or triplicate. Student *t*-test was used for statistical evaluation. The results were considered significant when p value was lower than. 0.05.

## Results

### Dynamic viscosity

The Dynamic viscosity measurements reported in Table 2 are represented as values extrapolated from the curves in the Newtonian plateau range (η_0_). Since it is known that the dynamic viscosity value of the stimuli is mainly ascribable to the H-HA fraction in the complex, we accomplished rheological measurements of H-HA 0.5%w/v and 0.05%w/v solutions. The highest value (378 mPa·s, Table 2) of η_0_ was found for H-HA 1 % w/v. The samples containing half this concentration reported a 7-fold lower η_0_ (55 mPa·s), whilst the complex formation with a global concentration of 1 % w/v (*i.e.* equal amount of the H-HA and L-HA) caused a η_0_ reduction of 3-fold and 21-fold (with respect to the H-HA 0.5%w/v and 1%w/v). These outstanding differences in rheological behaviour presumably could influence the friction experienced by cells during migration towards wound closure. The η_0_ values for the stimuli at 0.1 and 0.05 % w/v were lower and more comparable thus proving that the combination of high and low molecular weight at reduced concentrations has minor influence on fluido-dynamic and thus on motility.

**Table 2:** Dynamic viscosity (η_0_) of H-HA and H-HA/L-HA at 0.05, 0.1,0.5 and 1 % w/v calculated with a oscillatory rheometer, extrapolated in a shear rate ranging from 1 to 10 s^−1^, in the Newtonian range plateau

Samples (w/v) in DMEM medium supplemented with 1 % FBS	Dynamic viscosity (η) (mPa·s)
H-HA 0.05 %	1.73 ± 0.05
H-HA 0.1 %	2.55 ± 0.07
H-HA 0.5 %	55.2 ± 1.20
H-HA 1 %	378 ± 3.00
H-HA/L-HA COMPLEX 0.1 %	1.65 ± 0.04
H-HA/L-HA COMPLEX 1 %	18.0 ± 1.00

### Stability of H-HA, L-HA and H-HA/L-HA in the presence of BTH

Enzymatic digestion of H-HA/L-HA complexes with respect to those of H-HA or L-HA alone was studied to evaluate resistance (or stability) eventually conferred by the complex formation. The results confirmed that L-HA was not attacked by BTH while there are appreciable differences in the enzymatic depolymerisation of HA alone when compared to the H-HA fraction in the complex [31]. As described in methods section, hydrolyses were monitored by SEC-TDA analysis. In particular, in the first 2 h of incubation, the high MW reduction percentage (calculated as [(Mw_t0_-Mw_t_)/ Mw_t0_.] × 100), reached 44 % for H-HA alone and only 11 % for the complex (data not shown). Moreover, a peculiar software application of the Viscotek chromatographer allowed for the measurement of the H-HA fraction with MW > 1MDa and contemporarily the L-HA fraction with Mw inferior to 200 kDa (%L-HA < 200 kDa) on samples withdrawn during BTH incubation. After 24 h, the data showed that the H-HA fraction with MW > 1MDa decreased by 50 % in the pure H-HA solutions while only 6.25 % reduction was found for the H-HA/L-HA complex. Contemporarily, L-HA% < 200 kDa proved relatively invariable (1.44 % of reduction) for H-HA/L-HA, while in the pure H-HA samples the low molecular weight fraction increased by 128 % (Table 3).

**Table 3:** Data extrapolated from Viscotek analysis of experiment concerning the degradation of H-HA, L-HA and H-HA/L-HA complex in presence of BTH at 24 h. The decrease percentage of HHA and of LHA analyzed through the software (Δ%HHA > 1000 kDa and %LHA <200 kDa) were calculated as the (Mw fraction at t_0_-Mw fraction t_24h_/Mw fraction at t_0_)

Sample	Δ %HHA > 1MDa	Δ %LHA <200 kDa
LHA	N.D.	N.D.
HHA	50	128
H-HA/L-HA	6.25	1.44

### *In vitro* scratch-wound healing assay

**Fig. 1:**
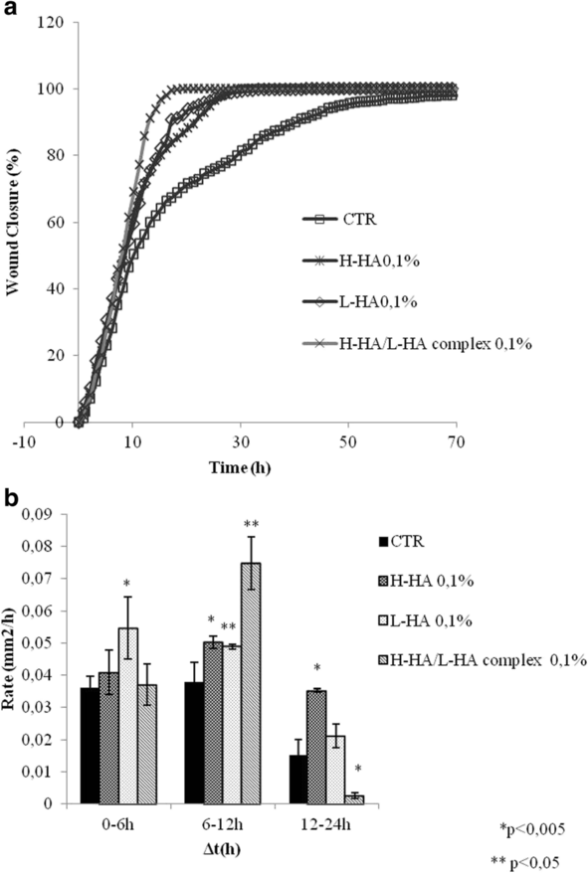
**a** Repair area percentage [as (*Areat*_0_ − *Area t*/*Areat*_0_) × 100] for the control and in presence of following stimuli: H-HA, L-HA and of H-HA/L-HA complex, all at 0.1%w/v; the curves are averages of three different experiments with variations within 5 % of the value. **b** Comparison of the healing rates between control and the samples H-HA, L-HA and H-HA/L-HA complex (0.1%w/v). The data represent an average of three repeats; with the terms repeats, we indicate that samples are collected from threeindependent experiments; data shown are means ± SD; the t student is calculated respect to the control (*p < 0.005; **p < 0.05)

**Fig. 2:**
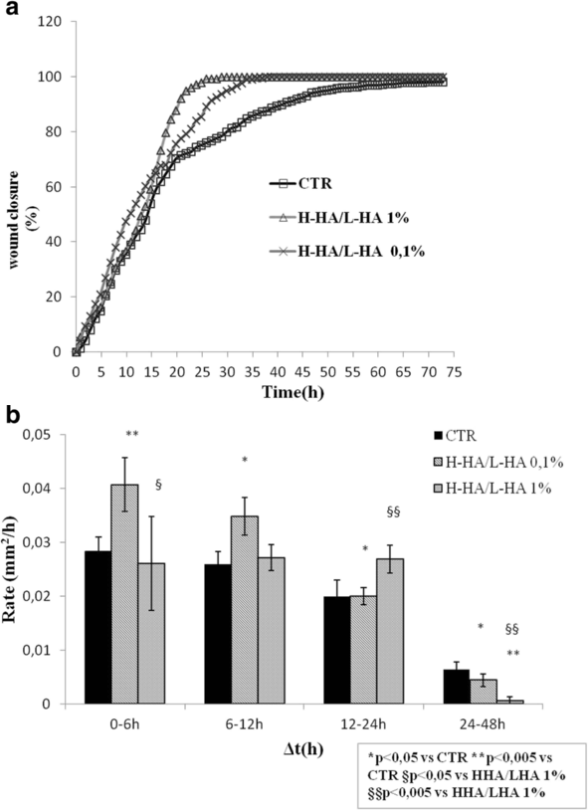
**a** Repair area percentage [as (*Area t*_0_ − *Area t*/*Area t*_0_) × 100 for the control and in presence of following stimuli: H-HA/L-HA complex at 0.1 and 1%w/v; The curves are averages of three indipendent experiments with standard deviation within 5 % of the value. **b** Comparison of the healing rates between control and the samples H-HA/L-HA complex 0.1–1%w/v). The data represent an average of three repeats; with the terms repeats, we indicate that samples are collected from threeindependent experiments; data shown are means ± SD; the t student is calculated respect to the control(*,**) and HHA/LHA 1 % (§,§§).(*p < 0.05; **p < 0.005; §*p* < 0.05, §§*p* < 0.005)

We investigated the effect of H-HA, L-HA and the H-HA/L-HA complex on wound closure in HaCaT cell monolayers. The results were reported in Fig. 1. It is evident that the scratch closure occurred at a faster rate in the presence of the diverse HA compared to the one in the control. Among the different HA gels analysed, the 0.1 % w/v H-HA/L-HA complex acted faster than either H-HA alone or L-HA alone, which were similar in their scratch closure rate. We derived the percent wound closure curves by averaging at least five fields from each triplicate independent experiment (Fig. 2a). At 20 h of culture, wound closure in the control was less than 75 %, while it had already reached 85–100 % in the presence of HA. Total wound closure occurred at 26 ± 2 h in the presence of 0.1 % H-HA or 0.1 % L-HA, and at 18 ± 1 h with 0.1 % H-HA/L-HA, suggesting a synergistic effect between the HA forms (Fig. 1a). Considering the rate of closure, HA and control cultures were mainly similar at 0–6 h, whereas better short-term (6–12 h) actions were evident for all the HA treatments, especially when the 0.1 % H-HA/L-HA complex was used (Fig. 1b).

Higher concentration of H-HA/L-HA complex (*i.e.*, 1 % w/v) was then tested to verify whether it was possible to further hasten wound repair. For this, a wider scratch (>1 mm) was manually inferred to the cell monolayer. Wound closure was quicker by almost 5 h with 1 % H-HA/L-HA (Fig. 2a). The 11-fold higher viscosity of the 1 % H-HA/L-HA complex with respect to the 0.1 % one (Table 2) initially hampered cell migration but later on, in the repair process, the mixture probably became less viscous due to degradation of H-HA, and enhanced reparation (Fig. 2b).

**Fig. 3:**
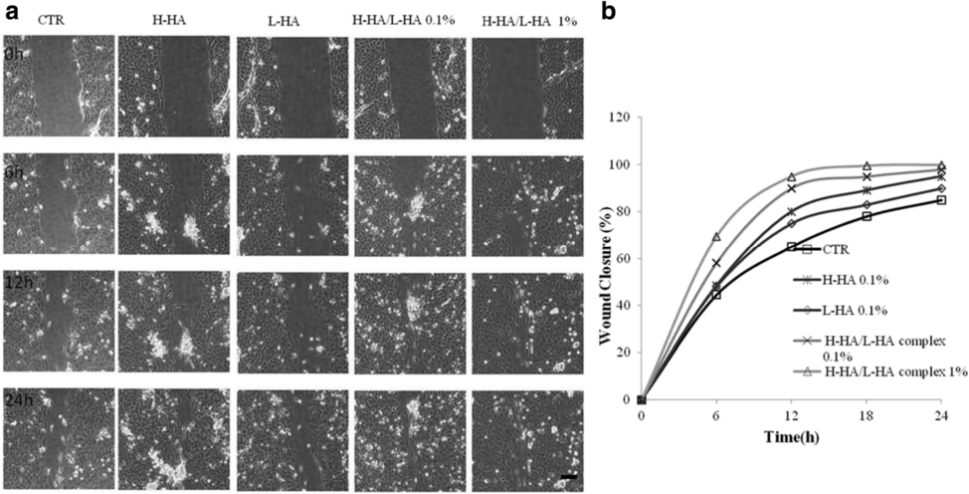
**a** Representative micrographs pictures of HaCaT-HDF scratch assays immediately after the scratches, and in time course of the experiments. Scale bar, 100 μm. **b** Repair area percentage [as (*Areat*_0_ − *Area t*/*Areat*_0_) × 100] for the control and in presence of following stimuli: H-HA, L-HA H-HA/L-HA complex at 0.1 and H-HA/L-HA 1%w/v; the curves are averages of three different experiments with standard deviation within 5 % of the value

Concerning the co-culture experiments, Fig. 3a showed representative images of HaCat and HDF cell migrating to repair the scratch in the control and in the presence of stimuli; we confirmed once more, better and faster reparatory effect of complexes respect to the single HA samples at both concentrations: 0.1 e 1 % w/w. Particularly, in the Fig. 3b, it is evident that the wound closure (%) in presence of H-HA/L-HA was 1.5 and 1.4 fold higher with respect to the control, while for HHA and LHA it improved ≈ 1.2 fold.

### Gene expression analysis

**Fig. 4:**
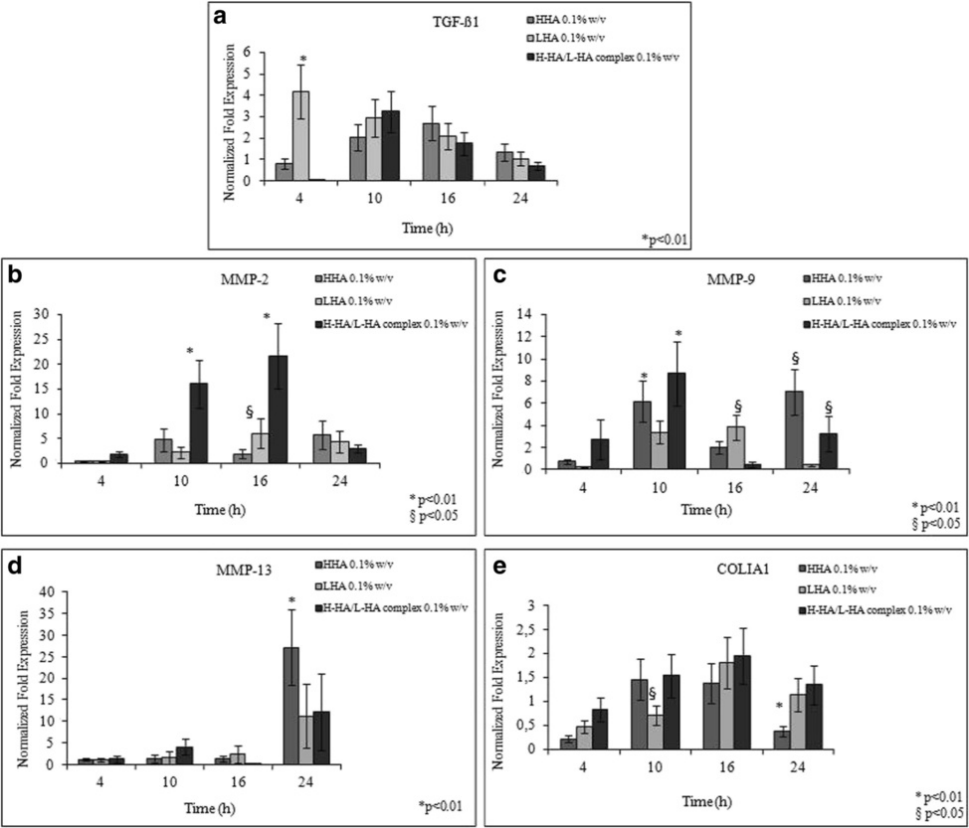
Gene expression analysis. The graphs showing normalized fold expression for the genes: transforming growth factor beta 1 (**a**), metalloproteinase-2 (**b**), metalloproteinase-9 (**c**), metalloproteinase-13 (**d**) and type I collagen (**e**). Data represent the mean ± SD of six independent experiments. The t student is evaluated respect to the control (*p < 0.01), (§*p* < 0.05)

The expression of several genes that are known to be regulators of epithelial cell migration during normal wound repair [32] was evaluated by qRT-PCR. The first marker analysed was TGF-β1. As a growth factor that performs different activities in proliferation and migration, it can also regulate MMP gene expression [33]. RT-PCR analyses showed that TGF-β1 was down-regulated at 4 h in cultures treated with 0.1 % w/v H-HA or 0.1 % w/v H-HA/L-HA complex, whilst it was not treated with 0.1 % w/v L-HA. In the presence of either the complex or H-HA, TGF-β1 expression was increased at 10 h and 16 h and, then, they became comparable to the control in the following 24 h. With 0.1 % w/v L-HA, TGF-β1 was up-regulated 4-fold with respect to control at 4 h, but decreased with increasing incubation time, maintaining similar behaviour to other samples for longer time intervals at 10, 16 and 24 h (Fig. 4a). The expression of the gelatinases MMP-2 (gelatinase A) and MMP-9 (gelatinase B), which are known to be implicated in regulating epithelial cell migration [34], were also evaluated. Both presented a bell-shaped expression trend in all treatments tested. Particularly, in the H-HA/L-HA complex-treated samples, maximal up-regulation for MMP-9 was observed after 10 h, while up-regulation of MMP-2 was postponed to 16 h (Fig. 4b,c). The H-HA- and L-HA-treated samples had a low MMP-2 expression at the early stage (4 h), followed by up-regulation (*i.e.* 10 h, 16 h e 24 h) in all the different treatments (Fig. 4b,c). This is consistent with an early cellular response prompted by insult-driven signalling, leading towards complete repair within 24 h. The effect was more evident with the 0.1 % w/v H-HA/L-HA complex, since MMP-2 expression was 16- and 22-fold higher than the control at 10 h and 16 h, respectively (Fig. 4b). In addition, MMP-13, which degrades interstitial collagen I-II and III [35], was increasingly up-regulated from 4 h up to 24 h (Fig. 4d). In particular, in the H-HA-stimulated cells, MMP-13 was markedly more expressed, specifically 27-fold *vs.* the control, and 15-fold *vs.* the other samples. Type I collagen gene expression was down-regulated at 4 h (Fig. 4e); however, later on, it presented an increment of up to 2-fold (16 h), with a slight reduction in expression at 24 h. This reduction was significant only for cells treated with H-HA solutions (Fig. 4e). With higher H-HA/L-HA complex concentration, TGF-β1 was down-regulated up to 16 h, and slightly up-regulated at 24 h (Table 4). Type I collagen was up-regulated in the presence of 0.1 % w/v H-HA/L-HA between 10 and 16 h, but only at 16 h with the higher complex concentration. Type I collagen then became markedly less expressed at 24 h for both the concentrations. This last result was expected in HaCaT cells in the last stage of the reparative process.

**Table 4:** Gene expression analysis: Normalized fold increase of H-HA/L-HA complex 0.1 and 1 % (w/v), relative to gene expression transforming growth factor beta 1 (TGF-β1) and Type I collagen (COLIA1) at different time of analysis. Data represent the mean ± SD of three independent experiments

	TGF-β1		COLIA1	
H-HA/L-HA complex (% w/v)	0.1	1	0.1	1
4h	↓↓	↓	**~**	=
10 h	↑	↓	↑	↓
16 h	↑	↓	↑	↑↑
24 h	=	=	=	↓

Legend: **=** as the control, **↑** 1.2–5 fold vs control, **↑↑** 5.2–10 fold vs control, **↑↑↑** >10 fold vs control, ↓ 0.1–0.7, ~ 0.8–0.9 fold vs control, **↓↓** < 0.1 fold vs control

### Zymography and enzymatic activity

**Fig. 5:**
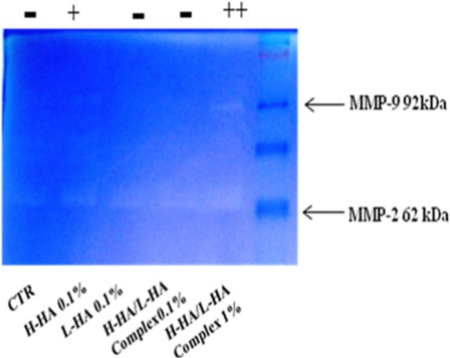
Gelatin zymography showing the activity of 62 kDa MMP-2 and 92 kDa MMP-9 presents in culture medium samples, CTR (untreated cells), H-HA 0.1 % (w/V) (1400 kDa), L-HA 0.1 % (w/V) (100 kDa), H-HA/L-HA complex 0.1 % (w/V) and H-HA/L-HA complex 1 % (w/V)

Zymography is a reliable and sensitive method to study proteolitic activity of MMPs. Our results showed the active form of MMP-2 (gelatinase A 72 kDa) and MMP-9 (gelatinase B 92kda). After 72 h, the MMP-2, 72 kDa form and the MMP-9, 92 kDa form, expression are up regulated in H-HA and H-HA/L-HA complex 1 % w/v, compared to the control (Fig. 5). Based on these findings, it is evident that H-HA and H-HA/L-HA complex 1 % w/v are efficient in increasing matrix degradation during wound repair, confirming the role of MMPs in this process.

The results of the activity assay based on gelatin to evaluate MMPs in the supernatant of the diverse cell cultivation, were reported in Table 5. The SEC-HPLC data, extrapolated, are represented as reduction percentage of the gelatin amount after 6 h of incubation time. The substrate degradation slightly increased in presence of the treatments respect to the control. However all the stimuli seemed to have a similar behavior in degrading the gelatin, except for the complexes 1 % that showed higher Gn reduction equal to 64 %, presumably corresponding to an increase of enzymes amount.

**Table 5:** MMPs enzymatic assay of cell medium un-treated (CTR) and treated (H-HA0.1 %, L-HA0.1 %, H-HA/L-HA 0.1 % and 1 %) after 24 of incubation with a scratched HaCat monolayer. The assay was performed for 6 h under stirring at 37 °C. The reduction percentage was calculated as [(Gn_t0_- Gn_t6h_)/Gn_t0_]*100 where Gn_t0_ is gelatin amount at initial time and Gn_t6h_ is gelatin amount after 6 h incubation time

Sample	Reduction percentage (%)
Gn + cell medium(CTR)	50,9
Gn + cell medium(H-HA 0,1 %)	57,6
Gn + cell medium(L-HA 0,1 %)	56,9
Gn + cell medium(H-HA/L-HA 0,1 %)	59,8
Gn + cell medium(H-HA/L-HA 1 %)	64,4

### Western blot and immunohistochemical analysis

**Fig. 6:**
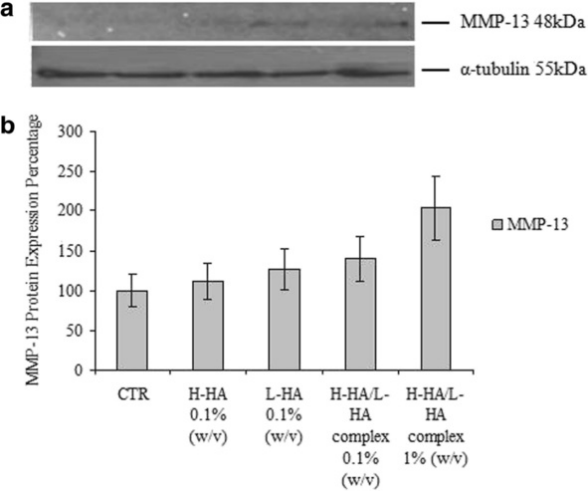
**a** Representative western blot analysis of metalloproteinase-13 (MMP-13) protein and α-tubulin housekeeping protein levels in the HaCat scratched cells at 16 h after scratch and different stimuli. **b** Western blot band densitometry: the histograms indicate the percentage variations in MMP-13 protein levels in the control and in treated cells compared to the un-treated cells (CTR). Replicates on each cell extract were run two different western blots. Data shown are means ± SD; the t student is calculated respect to the control and H-HA/L-HA complex 0.1 and 1 % (**p < 0.01)

**Fig. 7:**
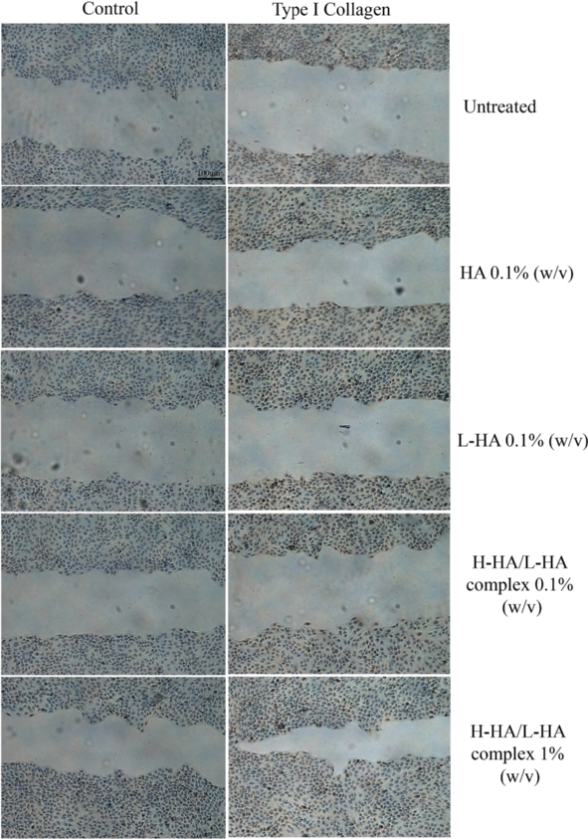
Immunohistochemical analysis for type I collagen (COLIA1) antigen on HaCat scratched cells at 12 h after treatment with different HA gels

**Fig. 8:**
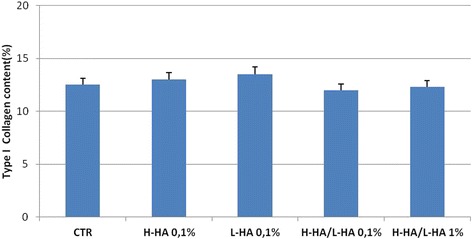
Quantitative analyses of Immunohistochemical for type I collagen (COLIA1) antigen on HaCat scratched cells at 12 h after treatment with different HA gels

Western blot analysis revealed a statistical and significant increase in MMP-13 protein level in the 0.1 % H-HA/LHA- and 1 % H-HA/LHA-treated samples compared with control, H-HA- and L-HA-treated ones (Fig. 6). Qualitative analysis of type I collagen revealed no net differences between the control and HA-treated cultures Immunohistochemical analyses showed positivity of the cells to type I collagen, but even using ImageJ analyses quantitative differences cannot be outlined (Figs. 7 and 8).

## Discussion

The assessment of robust and efficient *in vitro* tests may contribute to diminish pre-clinical studies on animals, a target that is endorsed by public opinion more and more. Bearing this in mind, the time-lapse video-microscopy station, and, developed assays in particular, may strongly contribute to simultaneous evaluation of biomolecules and their analogues. The use of this technique in wound healing studies is nowadays well established and reports in the literature are copious [36–39]. This has previously been used to obtain evidence that hyaluronic acid and its fragments play a key role in the re-epithelization process [6]. The present model is based on HaCaT cells because keratinocytes are known to migrate from the basal layers of the epidermis to a more superficial one triggering an array of biochemical events that lead towards the reconstitution of full-thickness skin [40]. All these events are modulated by HA [28].

Generally we can assess that HA based commercial products contain higher amounts of hyaluronic with respect to amounts present in physiological conditions (skin ([HA] = 0.5 mg/g wet tissue) because these were thus diluted in the skin or used for other treatments [28].

At concentrations comparable to commercial products, high- and low- molecular weight hyaluronans were compared and evaluated, with respect to novel, tailor-made complexes constituted from a mixture of these two components, for their stability in hyaluronidase attacks and their effect on scratch-wound healing in *in vitro* assays. Interestingly, H-HA/L-HA complexes proved to have better resistance to hyaluronidase (BTH). In fact, the lower molecular weight fraction release was reduced compared to H-HA samples during enzymatic digestion. This behaviour supported the idea that this novel species has a non-detectable conformation for the enzyme and thus is more resistant for application in *in vivo* tissue. Hyaluronidase seemed to be less active on H-HA present in these hybrid complexes with respect to H-HA alone. Furthermore, with regards to L-HA degradation, it must be highlighted that its enzymatic digestion is generally slower than H-HA [31] and therefore in our opinion less interesting and in our results, because degradation is inexistent when L-HA chains were entrapped in the hybrid complexes. However this result is in accordance with the lower activity of BTH on smaller HA chains (*e.g.* in the conditions used in this work (0.5 U/ml) e are not able to degrade LHA <100KDa).

We found that presence of H-HA and L-HA forms improve the reparative process, shortening the closure time of the scratch. The reparative rate depended upon both HA treatments used and the time elapsed. In the first few hours (0–6 h) of healing, L-HA (90 kDa) was more effective than either H-HA or the 0-1 % H-HA/L-HA complex and stimulated TGF-β1 gene expression. Recent literature asserts that smaller hyaluronan molecules (usually with an average molecular weight ranging from 5 to 20 kDa) are often promoters of early inflammatory responses, which activate signalling cascades to prompt cell migration and proliferation towards repair of a lysed area [41].

In spite of the great difference in molecular weight, in the medium time range (6–12 h), H-HA- and L-HA-treated cultures displayed a similar repair trend. This outcome could probably be attributed to the degradation kinetics of H-HA, which is faster than that of L-HA [31]. Moreover, H-HA/L-HA complexes acted as slow release systems, freeing L-HA earlier than H-HA, and thus prompting regeneration later on. With these complexes, scratch closure was completed within 18 h, one-fourth of the time needed for control cultures. Therefore, it can be argued that the combined effect of high- and low-molecular weight HA is fundamental for tissue repair. In particular, a slow release of L-HA at the beginning of incubation probably mimics the effect of slight inflammatory stimulus and may activate the biosynthesis machinery towards healing. This last phenomenon has been observed *in vivo* for H-HA and L-HA at the injury site [7–9]. These results were also corroborated by a statistically significant increase in the closure rate at the 6–12 h interval in the presence of H-HA/L-HA complex.

In addition, co-culture with human dermal fibroblast and keratinocytes [42] was accomplished in order to better mimic the actual *in vivo* situation and confirmed the best beneficial effect of complexes with respect to LHA, but even of HHA alone. Reparation occurred in a shorter time, with respect to keratinocyte monolayer probably due to the synergic effect of two different cellular populations, even if the curves showed similar trends to the ones obtained for single culture scratch monolayer.

Another interesting feature is the lower viscosity attained through complex formation, that does not occur when L-HA and H-HA are mixed after being autoclaved. In fact, generally, a viscous hydrogel may hamper cell migration. Surprising H-HA/L-HA complex behaviour permitted the use of a 1 % w/v H-HA/L-HA complex gel, which enhanced the bio-revitalizing effect. Although drop in viscosity was at least 10-fold of H-HA/L-HA with respect to the same H-HA amount, keratinocyte migration was initially hampered at 1%w/v concentration, as showed by the lower closure rate 0–6 h (Fig. 2). We can speculate that hyaluronidases produced by the cells degraded H-HA in this specific time frame thus decreasing viscosity. This bio-catalytic process leads to a great amount of hyaluronic fragments in the solution, accelerating the future reparative action (Fig. 2) [10, 14, 15, 17, 31].

In order to support the macroscopic and morphological results obtained through time-lapse experiments, we carried out molecular analysis of markers involved in the re-epithelization process of keratinocytes immediately after injury. The aim was to evaluate modulation of cellular repair mechanisms when stimulated with biologically active polymers like hyaluronan and the novel derived complex. TGF-β1 is among the growth factors that have a positive influence on re-epithelialization [43]. It is expressed in epidermal keratinocytes and increases during cutaneous wound healing, contributing to the regulation of motility and stimulating gene expression of ECM components, MMPs and integrins [44, 45]. Initially, TGF-β1 was down-regulated in H-HA/L-HA-complex treated samples (Fig. 4). Successively, gene expression was similar for both H-HA and the complexes, finally resembling control expression level at 24 h. Instead, cells treated with L-HA expressed high levels of TGF-β1 already at 4 h being between 4 and 2 fold higher that the control up to 16 h incubation. TGF-β1 overexpression could be responsible for the slight inflammatory response necessary in the recruitment of MMPs [26]. Different patterns of MMPs expression during the repair phase have been reported in *in vitro* models of HaCaT monolayers [46]. In our study, we found an up-regulation of MMP-2 and -9, especially between 4 and 16 h, when the fastest repair rate was observed. We hypothesize that the lowering in viscosity caused by complex formation modified shear forces and attrition around the cells, permitting faster cellular migration.

In addition, zymography results confirmed that gelatinases, besides the higher RNA expression, are produced and biologically active. Quantitative differences could not be evidenced due tho the technique itself. Therefore a quantitative SEC-HPLC based assay was assessed and Gn degradation was slightly superior in all the treated samples, the highest conversion been observed with H-HA/L-HA complex 1 %. The increases of the MMP2 and 9 expression in presence of treatments, and specifically with complexes was closely correlated with the increase of reparation rate.

To further investigate the involvement of the ECM in wound healing, we examined the expression of MMP-13. MMP-13 known to be expressed in immortalized human epidermal keratinocytes [45]. We found progressive up-regulation of MMP-13 mRNA up to 24 h. It is evident that cells cannot migrate through native type I collagen without degrading it, first by the action of MMP-1 and then by that of MMP-13 [45]. According to Hattori and collaborators [34], MMP-13 expression is higher in the early phase of wound repair for murine models. Conversely, we showed different gene behaviour in our human, transformed keratinocytes [45]. The presence of a higher MMP-13 expression was also supported by Western blotting analysis, suggesting enhanced degradation on ECM (an event required for repair). MMP-13 gene expression could be associated with its preferential substrate: type I collagen synthesis [46]. Regulation of collagen synthesis and its deposition control scar tissue formation. We found up-regulation over time of type I collagen, with a slight reduction at 24 h in all treatments. This could be consistent with collagen synthesis occurring at lower rates in later phases of wound regeneration, that is a necessary event for remodelling [47]. We assume that the gradual repair and re-building of cell–cell connections prompts collagen synthesis and MMP-13 activation. The main outcome is higher positivity for type I collagen in HaCaT, in the presence of 1 % w/v H-HA/L-HA complex, proving once more a close correlation between matrix formation and cell proliferation.

As shown in Table 3, when comparing different concentrations of the H-HA/L-HA complex, we found a down-regulation of TGF-β1 in the 0–16 h time range, confirming a minor inflammatory effect elicited in this specific model. Concerning the expression trends, the behaviour of COL1A1 in the presence of both H-HA/L-HA complexes was similar. The difference in gene expression between the two stimuli was detectable within 10–16 h. Results were also supported by wound-closure data in Fig. 3 demonstrating inverted reparative curves at around 14–15 h. Generally lower expression level of COL1A1 in HaCat rather than other cells (*e.g.* fibroblasts) was due to the different embryonic origin (ectodermal and mesodermal origin). The down-regulation of COL1A1 in the presence of 1 % H-HA/L-HA complex at 24 h can be probably ascribed to a general higher reparative rate that leads to earlier complete wound closure.

Overall, the empirical and qualitative evaluation of *in vitro* wound repair was corroborated by quantitative evaluation of TLVM devices and software, demonstrating the effectiveness of the assay. In our study, we did not observe any persistent inflammatory effect of the 90 kDa L-HA used, suggesting that controversial data in the literature should be strictly attributed to short HA fragments [7, 18]. Interestingly, H-HA/L-HA-complexes improved sole H-HA performance. The fact that TGF-β1 expression modulation was almost four times less for the complexes than for L-HA is very important, proving the entanglement of short chain with longer ones. Based on hydrogen bonding, this bridging looses small chains over time, releasing them to trigger the biochemical arrow towards the repair target.

Finally, the lower viscosity of the complexes permits delivery of a higher HA amount to the injury site, hastening the repair process.

## Conclusions

The concurrent analyses of TLVM and gene expression can contribute to extricate complicated cellular phenomenon and to evaluate the action of diverse biomolecules with the use of *in vitro* models. In this study, L-HA proved not to be toxic/inflammatory, and therefore permitted wound closure similarly to the well-known bioactive H-HA. Novel hybrid complexes formed by H-HA and L-HA performed better than HA alone, both at high or low concentrations. Complexes also showed better stability of long chains HA to hyaluronidases attack, presumably prolonging their half-lives *in vivo*. L-HA accelerates wound repair at an earlier stage, while H-HA has no short-term effect, probably due to its initial higher viscosity. The outcomes of this study may be the pillars for further *in vivo* studies to promote HA hybrid complex use in innovative medical devices for tissue regeneration.

## Abbreviations

HA: Na-Hyaluronate
H-HA: High molecular weight hyaluronic acid
L-HA: Low molecular weight hyaluronic acid
HaCaT: Human keratinocytes cell line
HDF: Haman dermal fibroblast
BTH: Bovine testicular hyaluronidase
TGF-β1: Transforming growth factor beta 1
EGF: Epidermal growth factor
TNFα: Tumor necrosis factor α
IGF: Insulin-like growth factor
MMPs: Metalloproteinases
COLIA1: Type I colllagen
ECM: Extracellular matrix
WH: Wound healing
PMN: Polymorphonuclear leukocyte
DMEM: Dulbecco’s modified eagle medium
FBS: Fetal bovine serum
PBS: Physiological buffer solution
TLVM: Time-lapse video microscopy
CO_2_: Carbon dioxide
RNA: Ribonucleic acid
cDNA: Complementary DNA
HPRT: Hypoxanthine-guanine phosphoribosyltransferase
NaCl: Codium chloride
EDTA: Ethylenediaminetetracetic acid
EGTA: Ethylene glycol tetraacetic acid
SDS: Sodium dodecyl sulphate
ECL: Enhanced ChemiLuminescence
qRT-PCR: Quantitative real time PCR
MMP-2: Gelatinase A metalloproteinase-2
MMP-9: Gelatinase B metalloproteinase-9
MMP-13: Collagenase metalloproteinase-13
HRP: Horseradish peroxidase
